# Cerebrospinal fluid/serum albumin ratio in patients with Lewy body disease: a systematic review and meta-analysis

**DOI:** 10.3389/fnagi.2024.1390036

**Published:** 2024-05-02

**Authors:** Moyu Li, Jinghuan Gan, Xia Yang, Shuai Liu, Yong Ji

**Affiliations:** ^1^Department of Neurology, Beijing Tiantan Hospital, Capital Medical University, Beijing, China; ^2^Department of Neurology, Beijing Friendship Hospital, Capital Medical University, Beijing, China; ^3^Department of Neurology, Tianjin Key Laboratory of Cerebrovascular and Neurodegenerative Diseases, Tianjin Dementia Institute, Tianjin Huanhu Hospital, Tianjin, China

**Keywords:** Qalb, Lewy body disease, Parkinson’s disease, blood–brain barrier, systematic review, meta-analysis

## Abstract

**Background:**

Abnormal cerebrospinal fluid (CSF)/serum albumin ratio (Qalb) levels have been observed in patients with cognitive impairment. Few studies have specifically focused on Lewy Body Disease (LBD), and the results were controversial. Thus, we conducted this systematic review and meta-analysis to investigate Qalb levels in patients with LBD by including data from different studies.

**Method:**

We systematically searched PubMed, Embase, Cochrane Library, and Web of Science databases for a collection of studies containing studies comparing Qalb levels in patients with LBD and healthy controls (including healthy controls and other dementia subtypes). In the initial search, 86 relevant papers were retrieved. Standardized mean differences (SMD) in Qalb levels were calculated using a random effects model.

**Results:**

A total of 13 eligible studies were included. Mean Qalb levels were significantly higher in patients with LBD compared to healthy older adults [standardized mean difference (SMD): 2.95, 95% confidence interval (CI): 0.89–5.00, *Z* = 2.81, *p* = 0.005]; and were significantly higher in patients with LBD than in patients with Alzheimer’s disease (AD) (SMD: 1.13, 95% CI: 0.42–1.83, *Z* = 3.15, *p* = 0.002);whereas mean Qalb levels were significantly higher in patients with frontotemporal lobar degeneration (FTLD) compared to those with AD (SMD: 1.13, 95% CI,0.14–2.13, *Z* = 2.24, *p* = 0.03).

**Conclusion:**

Qalb levels were significantly elevated in LBD patients compared with normal older adults and were higher than those in AD patients and FTLD patients, which helped in the differential diagnosis of LBD from other neurodegenerative diseases.

**Systematic review registration:**

https://www.crd.york.ac.uk/prospero/, identifier CRD42024496616.

## 1 Introduction

Lewy body diseases (LBD) are characterized by abnormal accumulation of α-synuclein (α-syn) to form Lewy bodies (LBs), including Parkinson’s disease (PD), Parkinson’s disease dementia (PDD) and dementia with Lewy bodies (DLB) (McCann et al., 2014). Most cases of LBD are related to age. So with the increase of life expectancy around the world, its prevalence rate is also increasing. About 11% patients with PD develop PDD every year, and the prevalence of PDD may be triple by 2060 (Savica et al., 2018; McCormick et al., 2019). The prevalence of DLB is also rising, accounting for about 5% of dementia. It is found that DLB is related to higher medical expenses and lower quality of life (Scarmeas et al., 2005; Mueller et al., 2017).

The blood–brain barrier (BBB) is a selective diffusion barrier that separates the central nervous system (CNS) from the peripheral blood circulation, which in turn restricts the entry of peripheral macromolecular proteins, cytotoxic substances, and peripheral immune cells into the nociceptors, maintains the stability of the internal environment of the CNS, and preserves the normal function of brain cells (Daneman and Prat, 2015). Disruption of the BBB allows the influx of neurotoxic blood debris, cells, and microbial pathogens into the brain and has been associated with inflammatory and immune responses, which may trigger multiple neurodegenerative pathways. BBB injury may disrupt metabolic homeostasis between vascular cells and brain cells, leading to problems with the control of the brain tissue’s exposure to blood-related substances, which may ultimately lead to cognitive impairment (Ryu et al., 2015). Often neuroimaging (Chagnot et al., 2021) and alterations in biomarkers (Wong et al., 2022) can be observed with BBB injury, suggesting an important role for the BBB in dementia (Raja et al., 2018). Recent evidence suggests that pathological forms of α-synuclein can regulate the expression of tight junction proteins that are essential for maintaining the BBB. This suggests that α-synuclein deposition may affect the integrity of the BBB (Bogale et al., 2021). BBB dysfunction has been associated with PD, particularly in the striatum, substantia nigra, and white matter of the brain (Burgmans et al., 2013), and is thought to play a role in disease progression and be involved in the development of cognitive impairment (Cai et al., 2018, 2021).

Current biomarkers used to identify impaired BBB integrity typically include serum levels of S100 calcium-binding protein B (S100B) as well as Qalb (Koh and Lee, 2014; Nation et al., 2019), which has appropriate but varying levels of sensitivity and specificity (Sun et al., 2021). Qalb has the advantage of being readily available as an easily accessible test in standardized automated clinical chemistry analyzers for routine use in general clinical laboratories worldwide. It is also the only biomarker for which fluids have been validated for clinical use (Skillbäck et al., 2017).

Although few studies have examined Qalb levels in LBD patients, there is an ongoing debate in the literature regarding the relevance of Qalb to the BBB in LBD patients (Kortekaas et al., 2005; Pisani et al., 2012; Janelidze et al., 2015; Page et al., 2021). To address this knowledge gap, we designed this systematic review and meta-analysis to summarize Qalb levels in patients with LBD, and we hypothesized that higher Qalb levels in LBD patients may be a reliable biomarker for distinguishing between LBD and AD/ frontotemporal lobar degeneration (FTLD).

## 2 Method

### 2.1 Data sources and literature search

This systematic review and meta-analysis used the Priority Reporting Items for Systematic Reviews and Meta-Analyses (PRISMA) guidelines. From the earliest available date to December 1, 2022, we conducted electronic literature searches using PubMed, Embase, Web of Science, and the Cochrane Library. Literature searches were conducted using the following search terms/keywords, but not limited to: (“Lewy body disease” OR “Lewy Body Dementia” OR “Diffuse Lewy Body Disease” OR“Cortical Lewy Body Disease” OR “Lewy Body Disease, Cortical” OR “Lewy Body Disease, Diffuse” OR “Lewy Body Type Senile Dementia” OR “Dementia, Lewy Body”) and (“albumin CSF/serum ratio” OR“cerebrospinal fluid/serum albumin ratio” OR “value of albumin quotients” OR “Qalb”). The searches were not restricted by age, race, or region. In addition, references in retrieved papers and reviews were manually searched for eligible studies. Two authors independently conducted literature searches and screened all titles, abstracts, and full texts of eligible studies. Disagreements were discussed with a third reviewer and resolved by mutual agreement.

**Figure 1:**
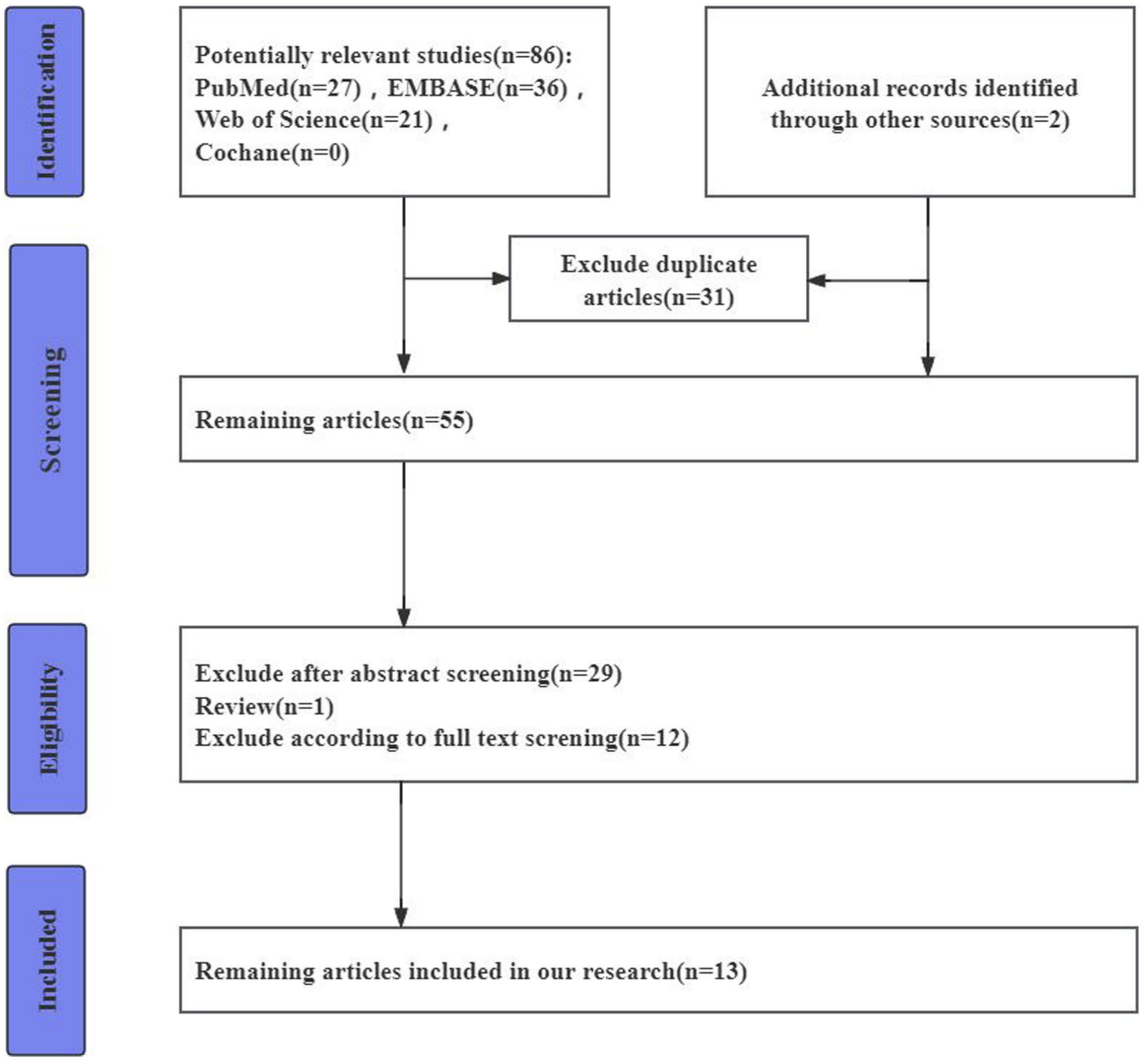
Flowchart of study selection.

The search and screening strategy and eligibility criteria based on the PRISMA guidelines are shown as (Figure 1).

### 2.2 Inclusion and exclusion criteria

Our analyses included studies that met the following criteria: (1) the primary eligibility criterion was to compare Qalb levels in patients with LBD and healthy controls/any other dementia subtype; (2) studies that provided a complete definition of participants. Diagnoses of different types of dementia/healthy controls were determined using clinical assessments and neuropsychological tests: AD was defined by the Diagnostic and Statistical Manual of Mental Disorders, Fourth Edition; International Statistical Classification of Diseases and Related Health Problems, 10th Edition, National Institute of Neurological Communicative Disorders and Stroke Criteria, and AD and Associated Disorders Association Criteria (McKhann et al., 2011). PDD was defined by the Parkinson’s Disease Society United Kingdom Brain Bank definition (Hughes et al., 1992). DLB was defined according to the DLB consensus criteria (McKeith et al., 2017). FTLD was defined according to the International FTLD Consensus Criteria (Neary et al., 1998); and (3) Qalb levels were measured and sufficient data (pg/dl) were available. (4) Language is limited to Chinese or English.

Similarly, studies were excluded if any of the following criteria were met: (1) there were no available Qalb data; (2) the publication was a letter, review, meta-analysis, or animal study; and/or (3) the Qalb assay methodology differed from the guidelines.

### 2.3 Quality assessment

The quality of the included studies was assessed according to the Newcastle-Ottawa Scale (NOS) criteria (Stang, 2010), including selection (0–4 points), comparability (0–2 points) and exposure (0–3 points). Scores ranged from 0 ~ 9, with no <7 indicating a high quality study. Disagreements were resolved by consensus and by seeking input from a third reviewer.

### 2.4 Statistical analysis

Data were analyzed using Review Manager 5.3 software. Since Qalb levels were continuous, standardized mean differences (SMD) were calculated and used for statistical analysis. The range between median and quartiles was converted to mean and standard deviation (SD) according to Hozo et al. (2005).

The funnel plot indicated the bias of the study; a symmetrical funnel plot was free of bias, and an asymmetrical funnel plot meant that bias was present. The *Z*-test was used to assess whether the results of the studies were significant or not, and a *p* value of <0.05 was considered significant, and vice versa. Heterogeneity between studies was assessed by Q value and *I*^2^, and the cut-off level of heterogeneity was set at 0.5. If *p* ≥ 0.05 or *I*^2^ ≤ 50%, the data were analyzed using the fixed-effects model; if *p* < 0.05 or *I*^2^ > 50%, the random-effects model was used. Subgroup analysis was used to explore inter-study heterogeneity, and sensitivity analysis was performed by removing studies one by one to explore the source of heterogeneity (Zhang et al., 2022).

## 3 Results

### 3.1 Study selection and characterization

We identified a total of 86 studies. After excluding duplicates (31 cases), a total of 55 studies were obtained. Of these studies, 1 study was excluded because it was a review (n = 1). Of the remaining 53 studies, 29 were excluded after reading the abstracts, whereas 13 studies were excluded after a complete review (Pirttila et al., 1994; Sjögren et al., 2000, 2002; Brettschneider et al., 2005; Nielsen et al., 2007; Boström et al., 2009; Janelidze et al., 2015, 2017; Llorens et al., 2015; Skillbäck et al., 2017; Bekris et al., 2018; Musaeus et al., 2020; Gan et al., 2023). These included 225 DLBs, 302 PDs, 1723 ADs, 190 FTDs, and 622 healthy controls(HCs). The age range of LBD patients is between 68 to 74 years, with males constituting 25 to 73 percent of the cases (Table 1).

**Table 1:** Characteristics of 13 studies included in meta-analysis.

Study	Year	LBD group				HCs group			
		Sample size	Age	Sex (M/F)	Qalb	Sample size	Age	Sex (M/F)	Qalb
Gan et al. (2023)	2023	19(DLB)	68.05 ± 9.48	10/9	6.31 ± 2.44	52(AD)	63.75 ± 9.85	21/31	7.24 ± 5.11
						24(FTLD)	67.38 ± 8.18	10/14	7.71 ± 3.77
Musaeus et al. (2020)	2020	28(DLB)	73.21 ± 5.92	7/21	9.60 ± 5.36	35(HC)	65.94 ± 7.53	14/21	5.47 ± 1.87
						289(AD)	72.64 ± 8.90	128/161	7.30 ± 3.57
						24(FTLD)	65.67 ± 10.50	12/12	7.09 ± 2.45
Bekris et al. (2018)	2018	13(PD)	NA	NA	1.00 ± 0.02	8(HC)	NA	NA	6.20 ± 2.20
Skillbäck et al. (2017)	2017	73(DLB + PDD)	73.00 ± 52.90	20/53	7.80 ± 3.90	100(HC)	73.00 ± 5.00	60/40	5.90 ± 2.20
						666(AD)	75.00 ± 6.00	448/218	6.80 ± 2.70
						56(FTLD)	67.00 ± 10.00	29/27	6.50 ± 2.40
Janelidze et al. (2017)	2016	34(DLB)	72.00 ± 6.00	13/21	8.40 ± 4.60	65(HC)	75.00 ± 6.00	42/23	6.30 ± 2.90
						75(AD)	76.00 ± 7.00	51/24	7.60 ± 3.40
						41(FTLD)	72.00 ± 6.00	21/20	8.60 ± 5.50
Llorens et al. (2015)	2015	105(DLB + PD + PDD)	70.78 ± 11.36	33/72	10.60 ± 8.16	34(HC)	66.00 ± 9.50	15/19	6.23 ± 2.16
						90(AD)	72.80 ± 10.90	70/20	6.31 ± 2.81
						14(FTLD)	65.40 ± 14.30	6/8	7.58 ± 2.63
Janelidze et al. (2017)	2015	100(PDD + PDND)	72.30 ± 6.00	35/65	4.88 ± 0.49	38(HC)	65.40 ± 8.60	22/16	3.50 ± 0.38
Boström et al. (2009)	2009	29(DLB)	74.00 ± 7.65	12/17	8.20 ± 3.20	49(HC)	73.00 ± 6.89	15/34	7.40 ± 3.20
						174(AD)	74.00 ± 8.67	52/122	7.30 ± 2.40
Sjögren et al. (2002)	2002	23(PD)	70.70 ± 9.00	17/6	7.40 ± 3.00	32(HC)	71.50 ± 5.20	8/24	6.20 ± 2.60
						60(AD)	66.00 ± 7.80	27/33	6.40 ± 2.40
						17(FTLD)	62.40 ± 10.20	6/11	5.60 ± 1.90
Sjögren et al. (2000)	2000	15(PD)	69.90 ± 7.50	11/4	6.70 ± 2.40	17(HC)	68.60 ± 7.50	3/14	5.20 ± 1.80
						19(AD)	70.60 ± 5.30	9/10	5.80 ± 1.70
						14(FTLD)	65.10 ± 5.30	4/10	5.80 ± 1.80
Pirttila et al. (1994)	1994	20(PD)	NA	NA	6.73 ± 2.53	42(HC)	NA	NA	5.78 ± 1.37
						40(AD)	NA	NA	5.91 ± 2.32

Qalb levels were measured by pg/dL. Qalb, cerebrospinal fluid/serum albumin ratio; LBD, Lewy body disease; HC, healthy controls; AD, Alzheimer’s disease; FTLD, frontotemporal lobar degeneration.

### 3.2 Qalb levels in Lewy body disease vs. healthy controls

**Figure 2:**
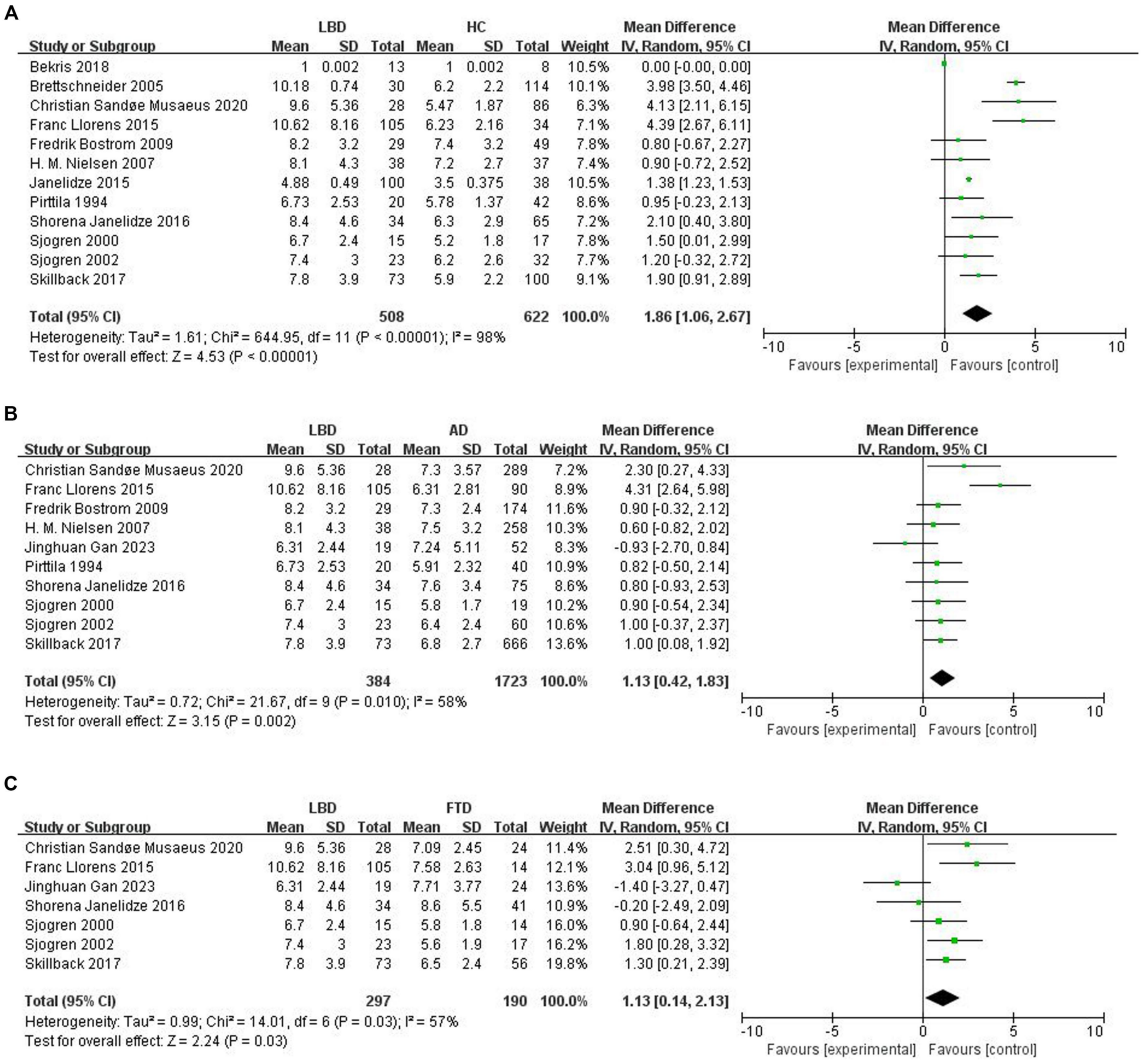
Forest plots of the Qalb in LBD compared with HC/other diseases. **(A)** Forest plot of Qalb in LBD compared with HC. **(B)** Forest plot of Qalb in LBD compared with AD. **(C)** Forest plot of Qalb in LBD compared with FTLD. CI, confidence interval; Qalb, cerebrospinal fluid/serum albumin ratio; LBD, Lewy body disease; HC, healthy controls; AD, Alzheimer’s disease; FTLD, frontotemporal lobar degeneration. SMD, standardized mean difference.

To compare Qalb levels in LBD patients with HCs, 12 studies were included (508 LBD patients, 622 HCs). The studies showed high heterogeneity (*I*^2^ = 98%, *p* < 0.00001), so a random-effects model was used to calculate the pooled SMD. The forest plot showed statistically significant higher mean Qalb levels in LBD compared with HCs (SMD: 1.86, 95% CI: 1.06 to 2.67, Z = 4.53, *p* < 0.00001; Figure 2A).

### 3.3 Qalb levels in Lewy body disease vs. Alzheimer’s disease

This part of the meta-analysis included 10 studies. The number of patients with LBD and AD was 384 and 1723, respectively. Study heterogeneity was high (*p* = 0.010, *I*^2^ = 58%), so a random-effects model was used to calculate the pooled SMD. A forest plot showed that Qalb levels were significantly higher in patients with LBD compared to patients with AD (SMD: 1.13, 95% CI: 0.42 to 1.83, *Z* = 3.15, *p* = 0.002; Figure 2B).

### 3.4 Qalb levels in Lewy body disease vs. frontotemporal lobar degeneration

Seven studies were included in this part of the meta-analysis. There are 297 and 190 patients with LBD and FTLD, respectively. Study heterogeneity was high (*p* = 0.03, *I*^2^ = 57%). The forest plot showed that Qalb levels were significantly higher in patients with LBD compared with those with FTLD (SMD: 1.13, 95% CI,0.14 to 2.13, *Z* = 2.24, *p* = 0.03; Figure 2C).

### 3.5 Heterogeneity analysis

#### 3.5.1 Sensitivity analysis

Sensitivity analyses were performed using the sequential removal of each individual study from the meta-analysis and observing the change in the combined effect sizes of the remaining studies. The results of the sensitivity analysis of Qalb level of LBD showed that the SMD value varied from 1.58–2.08, the lower limit of 95% CI was ≥0.59, the *Z* value fluctuated from 2.81–4.54, and the I2 fluctuated from 92 to 98%, and the *p* value was consistently less than 0.005, which indicated that the results of this study included in the literature were stable, and were not overly influenced by any single study, and no statistical differences disappeared or reversed.

#### 3.5.2 Subgroup analysis

The prevalence of DLB can be found to be higher in males than females in the previous studies (Musaeus et al., 2020). Therefore, in this paper, subgroup analyses of the proportion of males with LBD (>50% males and ≤ 50% males) were performed separately, and sex was not found to be a source of heterogeneity (Hughes et al., 1992; Table 2).

**Table 2:** Subgroup analysis.

Subgroup	Study	Sample size	SMD(95%CI)	I^2^(%)	Z-value(*p*-value)
Percentage of males>50%	2	38	1.35(0.29–2.42)	0	2.50(P = 0.01)
Percentage of males<50%	6	407	2.03(1.22–2.85)	72	4.87(P<0.00001)

#### 3.5.3 Publication bias analysis

**Figure 3:**
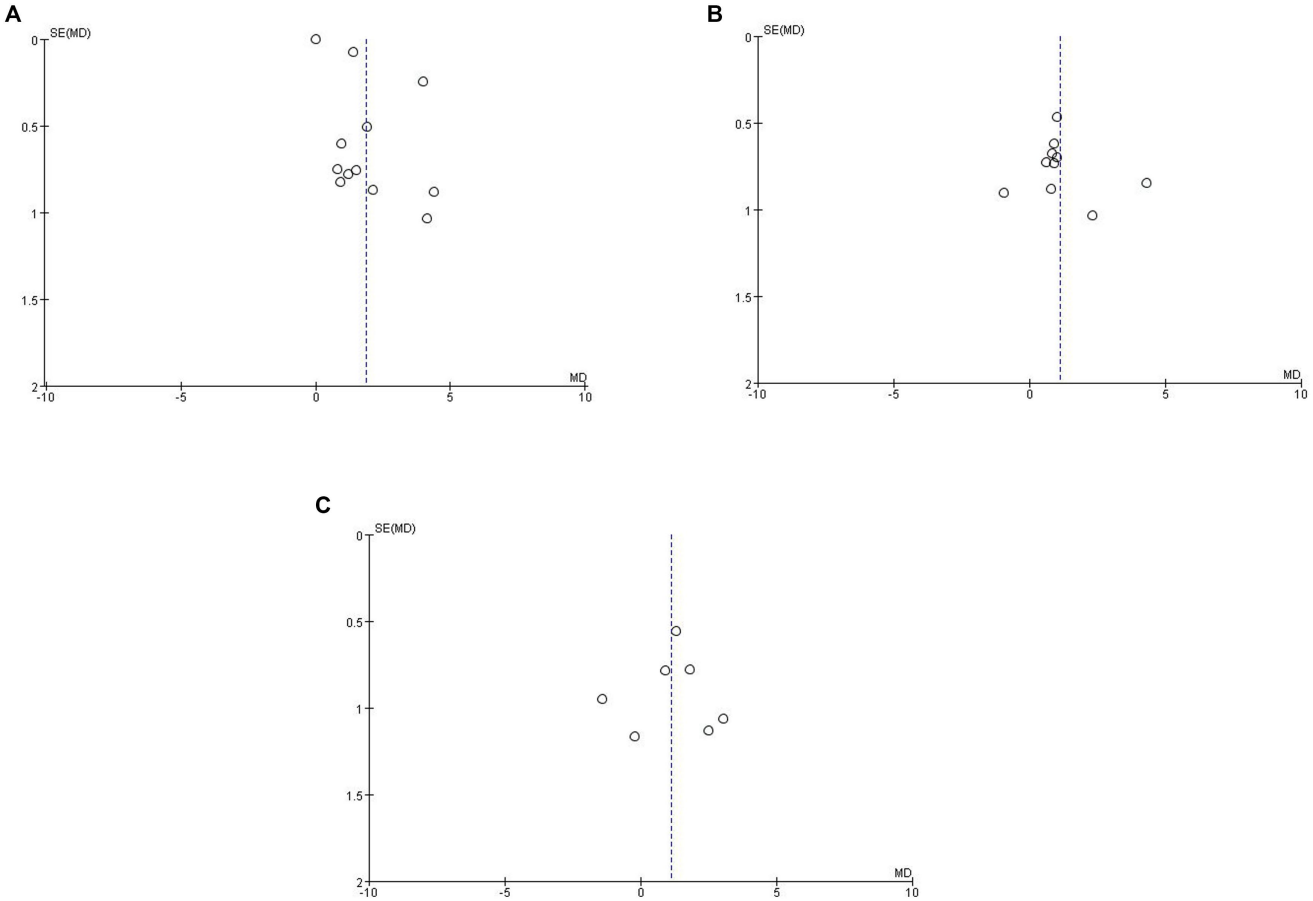
Funnel plot of Qalb in LBD compared with HC/other diseases. **(A)** Funnel plot of Qalb in LBD compared with HC. **(B)** Funnel plot of Qalb in LBD compared with AD. **(C)** Funnel plot of Qalb in LBD compared with FTLD. Qalb, cerebrospinal fluid/serum albumin ratio; LBD, Lewy body disease; HC, healthy controls; AD, Alzheimer’s disease; FTLD, frontotemporal lobar degeneration; SMD, standardized mean difference.

A funnel plot was made for each of the 13 selected studies to assess publication bias, and publication bias was assessed by visual inspection of the funnel plots for each analysis. The points in Figures 3A,C were evenly scattered on both sides of the midline and were basically symmetrical, which was considered to be unlikely to cause publication bias, while the points in Figure 3B were scattered on both sides of the midline but were unevenly distributed, which was considered to be possibly due to the small sample size.

## 4 Discussion

We demonstrated the potential role of Qalb levels in the differential diagnosis of LBD and other diseases. In this meta-analysis, which was performed by screening the literature, Qalb was significantly higher in patients with LBD compared to healthy controls and also compared to patients with AD and FTLD. Therefore, Qalb can be used as a diagnostic aid for LBD and a reliable biomarker to differentiate between LBD and AD/ FTLD, as well as to support clinical and neuroimaging findings to improve the accuracy of clinical diagnosis of these disorders.

In previous studies, alterations in Qalb have been recognized as a reliable standard surrogate marker of BBB integrity and a potential biomarker for neurological disorders (Wong et al., 2022). Qalb within a threshold range indicates “normal” CSF permeability, whereas exceeding the threshold reflects CSF circulatory dysfunction (Tibbling et al., 1977). There are conflicting reports on whether Qalb varies between different dementia subtypes. There are published reports of increased Qalb compared with healthy controls in patients with AD, DLB, and FTLD (Janelidze et al., 2015; Llorens et al., 2015; Skillbäck et al., 2017), as well as reports in which no difference in Qalb was found compared with healthy controls (Bien-Ly et al., 2015; Olsson et al., 2016). Differences may be due to differences in patient numbers, mean patient age, and cognitive function.

### 4.1 Qalb levels in Lewy body disease vs. healthy controls

In the present study, we compared Qalb in LBD and healthy controls. We observed that Qalb levels were significantly higher in patients with LBD, with a significant difference compared to healthy controls (*p* < 0.05).DLB disease and Parkinson’s disease are the two main forms of LBD, sharing common clinical and neuropathological features (Wakabayashi et al., 2013). Previous studies have published reports that Qalb in DLB is increased compared with the healthy controls (Janelidze et al., 2015). Several important cerebrospinal fluid biomarker studies have reported elevated Qalb in patients with early PD compared to the healthy controls (Janelidze et al., 2015; Liguori et al., 2017; Page et al., 2021). These studies are consistent with our analysis, which suggests that impairment of the BBB may be present in patients with LBD.

Neuroinflammation is an important factor in the onset and progression of most neurodegenerative diseases. Alterations in BBB permeability caused by the release of proinflammatory cytokines directly or indirectly contribute to glutamate excitotoxicity, astrocyte and microglia activation, and free radical production (Cheng et al., 2016). Neuroinflammatory cytokines in blood, serum, cerebrospinal fluid, and brain tissue have been shown to distinguish between different types of synucleinopathies (Wang et al., 2023). In patients with LBD, neurologic damage is accompanied by an inflammatory response, which may lead to increased permeability of the BBB, allowing proteins in the cerebrospinal fluid to penetrate into the bloodstream, which in turn raises Qalb levels.

### 4.2 Qalb levels in Lewy body disease vs. Alzheimer’s disease

Significant differences (*p* < 0.05) were observed when comparing Qalb levels in patients with LBD and AD, which may result from differences in BBB permeability between the two diseases. However, other mechanisms and influences may also be involved in the regulation of Qalb levels. It is suggested that Qalb may differ in the pathogenesis of AD and LBD.

AD and DLB are the most common neurodegenerative dementias, and both are characterized by β-amyloid (Aβ) misfolding and Aβ peptide deposition in the senile plaques (Aβ deposition) (Glenner et al., 1984). Musaeus et al. demonstrated that Qalb was higher in DLB patients than in AD patients (Musaeus et al., 2020). DLB is characterized by the deposition of 300 Lewy bodies, which are intraneuronal abnormal deposition of Aβ (Capouch et al., 2018). The cause of the accumulation of Lewy vesicles is largely unknown, but studies have shown that high concentrations of albumin increase fibrillation of α -synaptic nuclear proteins, which induces the formation of Lewy vesicles (Uversky et al., 2002; Munishkina et al., 2004). It has been hypothesized that albumin in the CSF of patients with DLB not only measures BBB permeability, but may be part of the mechanism of Lewy body formation. Two studies have found that human plasma albumin (HSA) significantly reduces α-syn aggregation and fibrillation, and it has even been proposed that HSA has a chaperonin-like ability to resist amyloid aggregation, which is the key to AD pathology (Kakinen et al., 1860; Bellomo et al., 2019). It is generally believed that damage to the BBB in patients and mouse models of AD accelerates the onset or progression of the disease, but the results of one study suggest that the BBB remains intact in a variety of preclinical models of AD (Bien-Ly et al., 2015). More research is needed to verify which explanation reflects the results of this study regarding Qalb in the LBD.

### 4.3 Qalb levels in Lewy body disease vs. frontotemporal lobar degeneration

We found that Qalb levels were higher in patients with LBD than in patients with FTLD (*p* < 0.05). One possible mechanism is the difference in the pathologic process of different types of dementia diseases. LBD and FTLD differ in the characterization of neurological damage, which may lead to changes in BBB permeability and thus affect Qalb levels.

In addition, age, disease duration, and severity of disease may also be influential factors in differences in Qalb levels. Longer disease progression, higher age, and greater severity of disease may result in more pronounced BBB damage, which in turn may affect Qalb levels.

FTLD disorders are a group of clinically and pathologically distinct dementia syndromes. Frontotemporal dementia (FTD) is the second most common early-onset dementia with clinical manifestations of progressive behavioral changes, executive dysfunction, and language difficulties. The three clinical syndromes of behavioral variant FTD (bvFTD), semantic dementia (SD), and progressive non-fluent aphasia (PNFA) constitute FTLD.

Higher Qalb levels in DLB patients than in FTD were observed in a study by Skillbäck et al. (2017). This is consistent with our findings. A study of postmortem cerebral microhemorrhages in patients with FTLD detected by 7.0 T MRI found that the predominance of cortical microhemorrhages in AD and FTLD was similar to that in LBD suggesting a similar origin. They are not associated with associated cerebrovascular disease, but may be due to brain barrier disruption associated with the neurodegenerative process itself (De Reuck et al., 2012). In terms of the discriminatory potential of Qalb between patients with LBD and FTLD, we identified only two studies; therefore, further studies are needed before definitive conclusions can be made about the reliability of this biomarker.

### 4.4 Subgroup analysis

For many years, Qalb was considered to be only an age-related parameter, but more recently it has also been linked to sex (Cosgrove et al., 2007). Exploring sex differences in the brain may be the key to understanding the role of sex in susceptibility to neurological disorders because of sex differences in brain development, structure, and neurotransmission (McCarthy et al., 2012). The estrogen 17β-estradiol may drive differential expression of enzymes involved in BBB breakdown, ultimately leading to a protective effect on the BBB (Na et al., 2015; Gu et al., 2017; Castellazzi et al., 2018; Yin et al., 2018). It is evident from literature data that males have higher Qalb values than females that seems not dependent from the underlying pathology. Our research is limited by the lack of specific Qalb data of relevant populations (including groups according to gender, age, cognitive level, onset age and course of disease). Thus, this could be a limitation of the current study.

## 5 Conclusion

In conclusion, Qalb has provided exciting and promising results for the differentiation of dementias. We made a meta-analysis of the existing literature, included the screened and high-quality studies, and evaluated the level of Qalb in cerebrospinal fluid of DLB patients, as well as its differences with AD and FTLD. Although Qalb is invasive, it provides a more reliable basis for the diagnosis of DLB.

Our meta-analysis suggests that Qalb levels can be used as a reliable biomarker to differentiate between patients with LBD and AD/FTLD and has the potential to improve the accuracy of the clinical diagnosis of LBD. In the future, studies with larger sample sizes are needed to confirm and investigate the mechanisms supporting our findings.

There are some limitations in our current study. Our research is limited by the lack of specific Qalb data of relevant populations. It was not possible to complete the heterogeneity analysis with high confidence due to the lack of complete information from data extracted from literature. Heterogeneity analysis is not well completed due to the number of available literatures. After subgroup analysis, meta-analysis and sensitivity analysis, the source of heterogeneity has not been found, indicating that this may be related to the heterogeneity of demographic characteristics between patients and healthy controls, as well as to the different procedures and technologies followed by different research groups. Adopting widely standardized methods and applying the same standards in future research may help to reduce heterogeneity.

## Author contributions

ML: Writing – original draft. JG: Writing – original draft. XY: Writing – original draft. SL: Writing – review & editing. YJ: Writing – review & editing.
